# Use of randomisation in clinical trials: a survey of UK practice

**DOI:** 10.1186/1745-6215-13-198

**Published:** 2012-10-26

**Authors:** Gladys C McPherson, Marion K Campbell, Diana R Elbourne

**Affiliations:** 1 Health Services Research Unit, University of Aberdeen, 3rd Floor, Foresterhill, Aberdeen, AB25 2ZD, UK; 2 Medical Statistics Unit, London School of Hygiene and Tropical Medicine, London, WC1E 7HT, UK

**Keywords:** Survey, Randomisation, Minimisation

## Abstract

**Background:**

In healthcare research the randomised controlled trial is seen as the gold standard because it ensures selection bias is minimised. However, there is uncertainty as to which is the most preferred method of randomisation in any given setting and to what extent more complex methods are actually being implemented in the field.

**Methods:**

In this paper we describe the results of a survey of UK academics and publicly funded researchers to examine the extent of the use of various methods of randomisation in clinical trials.

**Results:**

Trialists reported using simple randomisation, permuted blocks and stratification more often than more complex methods such as minimisation. Most trialists believed that simple randomisation is suitable for larger trials but there is a high probability of possible imbalance between treatment groups in small trials. It was thought that groups should be balanced at baseline to avoid imbalance and help face-validity. However, very few respondents considered that more complex methods offer any advantages.

**Conclusions:**

This paper demonstrates that for most UK trialists the preferred method of randomisation is using permuted blocks of varying random length within strata. This method eliminates the problem of predictability while maintaining balance across combinations of factors. If the number of prognostic factors is large, then minimisation can be used to provide treatment balance as well as balance over these factors. However, only those factors known to affect outcome should be considered.

## Background

The randomised controlled trial is widely regarded as the principal method for obtaining a reliable evaluation of treatment effectiveness of healthcare interventions. Randomised controlled trials work on the principle of random allocation – that is, unbiased allocation means we can be confident that any observed differences between treatment groups are due to the treatments being examined rather than due to any inherent differences between the groups [1].

Random allocation to treatments is used to ensure that patients in each treatment group will have similar distributions of characteristics, permits the use of probability theory to express the likelihood that the response to treatment is due to chance, and should not allow the next treatment allocation to be known in advance of the patient's entry into the trial.

Randomisation is a method of eliminating bias in the way that treatments are allocated to patients, but it does not guarantee that the characteristics of the groups will be completely balanced; if imbalance does occur, however, it will have occurred by chance rather than by the introduction of some systematic bias [2]. Some factors are known in advance to be important risk factors associated with the outcome of the patient. Some researchers believe that imbalance in these prognostic variables could have a marked effect on the results of a trial and on their credibility, in that it may be difficult to attribute observed differences to the treatments under test. While it is possible to modify the statistical analysis to take account of any differences between groups at baseline, some authors have indicated it is preferable to control for potential imbalance at the design stage [3,4].

There are four main classes of randomisation procedure available to the trialist when designing a trial: complete (simple) randomisation, restricted randomisation, covariate-balancing randomisation and response-adaptive randomisation (Table 1). These classes all have different properties and influence treatment assignment in different ways. Despite this range, it is often not clear which method should be implemented for a specific trial.

**Table 1:** Four main classes of randomisation procedure

**Class**	**Procedure**
Simple randomisation	Each patient has a known chance, usually equal, of being given each treatment and the treatment to be given cannot be predicted in advance. The simplest method is tossing a coin, but one can also use random tables or a random number generator on a calculator or computer.
Restricted randomisation	Imposing specific constraints on the randomisation process (for example, random permuted blocks) with a view to ensuring balance in the number of patients allocated to each treatment.
Covariate-balancing randomisation	Often it is desirable not only to achieve similar numbers of patients in each treatment group but also to ensure that patient groups are similar with respect to important prognostic factors such as age or gender. A number of mechanisms have been put forward to ensure balance across important prognostic factors – the most common of these include stratification and minimisation.
Response-adaptive randomisation	The treatment assignments depend upon previous patient responses to treatment.

In 1997 Assman and colleagues evaluated the allocation methods used in a sample of 50 trials published in four leading journals (*British Medical Journal*, *The Lancet*, *Journal of the American Medical Association*, and *New England Journal of Medicine*) [5]. They found that 30% used random permuted blocks, 6% minimisation, 2% simple randomisation and 44% did not specify the method used. In 2000 Hewitt and Torgerson reviewed 232 trials published in the same four journals [6]. At that time 50% used permuted blocks, 7% minimisation, 9% simple randomisation and the method used was unclear in 34%.

The aim of this study was therefore to determine the most commonly used method(s) of randomisation among a population of UK academics and publicly funded researchers working in the field of clinical trials and to investigate the extent of use of dynamic allocation methods such as minimisation.

## Methods

### Survey population

The survey was sent out at two time points: the primary survey was sent out in 2003, and a follow-up survey was sent out in 2011 to assess for any changes in opinions and practice over time. The same survey instrument was sent out at both time points (see Additional file 1).

In 2003 the Directory of Academic Statisticians [7], which was the most comprehensive directory of statisticians and methodologists working in academic research in the UK at that time, was selected as the sampling frame for statisticians and researchers working in the field of clinical trials.

Within the Directory, people who identified themselves with the following categories of research interest were selected: health, healthcare, health service research, health statistics, health technique assessment, medical statistics, clinical trials and randomisation. The initial search yielded 527 people. The full text entry for each person was examined and this led to the removal of 158 people whose areas of expertise were subsequently determined to be not relevant – for example, decision support systems in the National Health Service; analysis of finance and health data; social statistician (crime, education, health, poverty, race) – resulting in a target survey population of 369. A further two statisticians who were known to have a specific interest in randomisation methods but who were not listed in the Directory were also included as part of the survey population. This gave a survey population of 371 that included known experts in the field and representatives from all major UK trials centres.

The questionnaire was sent out together with an accompanying letter explaining the purpose of the survey. The questionnaire was printed on yellow paper as there was some evidence that paper colour could enhance response rates [8]. A reference number was included on the questionnaire in order to track responses, but this was removed by two people in order to preserve anonymity. Recipients were requested to pass the questionnaire to another researcher if it was not relevant to themselves. No reminders were sent out as the questionnaire was intended to be anonymous.

In January 2011 the survey was sent out a second time to see whether practice had changed in the intervening period. By 2011 the structure of trials support had changed radically in the UK with the establishment of formal clinical trials units, which are required to be registered with the UK Clinical Research Collaboration. Each trials unit has a named information specialist lead (who will generally be the first point of contact for randomisation services). Reflecting this change, the survey was this time sent (by email) to the information specialist for each registered trials unit. Questionnaires were sent to 48 registered trials unit statisticians (70 members) and information specialists (62 members) on the UK Clinical Research Collaboration mailing lists. As before, no reminders were sent to nonresponders.

### Data analysis

Data were analysed descriptively using numbers and percentages for binary variables. Responses to open-text boxes were categorised into common themes by the lead author (GCM). No ethical approval was required or sought.

## Results

Of the 371 questionnaires sent out in 2003, 185 (50%) were returned – 103 (56%) of these had some experience of setting up the randomisation for a clinical trial and are used in the analysis. Of the 132 questionnaires sent out in 2011, 15 (11%) questionnaires were returned. Results are presented for the combined survey population unless there were observed differences between the time points.

### Opinions about and use of different statistical randomisation methods

In 2003, 52% of respondents had used minimisation but stratification, permuted blocks and simple randomisation were more often used (Table 2). Many respondents used more than one method in different trials. In 2011 there was an increase in the proportion reporting having used minimisation (87%), comparable with stratification and permuted blocks.

**Table 2:** Use of different randomisation methods

**Method used**	**2003**	**2011**	**Total**
	(***n***= **103**)	(***n*** = **15**)	(***n*** = **118**)
Stratification	86 (84%)	13 (87%)	99 (84%)
Permuted blocks	82 (80%)	13 (87%)	95 (81%)
Simple randomisation	68 (66%)	10 (67%)	78 (66%)
Minimisation	54 (52%)	13 (87%)	67 (57%)
Other methods	10 (10%)	2 (13%)	12 (10%)

Data presented as *n* (%).

When asked which methods they would use for small trials, 61 (52%) indicated that they would use permuted blocks, 22 (19%) stratification and 30 (25%) minimisation. When asked about stratification, 43% wrote that the optimum number of strata was dependent upon the size of trial. For those willing to put a figure to this, the values ranged from 1 to 100 (interquartile range = 4, 9). Twenty per cent stated that the number of strata should be limited so that the expected number of participants in each stratum would not be too small: anywhere between 10 and 50 participants or 5 to 10% of cases. Only one person gave Therneau’s formula [9]:

$$\begin{array}{l} \text{No}.\text{of strata}<\text{N}/\text{blocksize}\;\text{and}\;\text{maybe}\;\\ \text{No}.\text{of strata}<\text{N}/\left(\text{blocksize}*2\right) \end{array}$$

Of those respondents who indicated that assignment of patients to treatment groups should take account of prognostic factors to show balance at baseline (*n*=67), the most common reasons given were that it avoids imbalance (*n* = 18) and that it helps face-validity (*n* = 13). Other reasons given were that it achieves a gain in efficiency (*n* = 5), achieves more precision in treatment effect estimate (*n* = 4) or that it allows for simple analysis later (*n* = 3). Those who disagreed that assignment should take account of prognostic factors (*n* = 18) maintained that adjusted analysis sorts out most of the imbalance, it is not necessary for large trials and that it is best left to chance.

### Opinions about and use of minimisation

Fifty-seven per cent of respondents had used the method of minimisation. Respondents indicated that the method seemed to work and most would use it again. The following methods were reported as being used to reduce the predictability of the next assignment: do not declare factors in the trial protocol; add in other irrelevant factors to provide noise; allocate the first block of about 20 patients (either some random number or 10% have been suggested) by permuted blocks or simple randomisation, and then use minimisation with a randomisation ratio of 0.75:0.25 (or 80/20); use blocking, and then restricted randomisation within each block; use centre as a minimisation factor in multicentre trials; use a probability between 0.5 and 1 for allocation; or alternate between simple randomisation/minimisation for small open trials.

When asked what was an appropriate level of randomness to use in minimisation, most respondents suggested a probability of from 0.66 to 0.95, with most saying that this level should be fixed throughout the life of the trial. Twenty-two respondents reported using values between 0.75 and 0.95, and seven had used values lower than 0.75. Seventeen respondents always used the method of Taves [10] with probability of assignment *P*= 1. Only one person always retained a random element in minimisation. One person had conducted a trial where the level of randomness was not fixed, but four others thought that it might be a good idea to vary randomness.

When asked about the number of factors that would sensibly be included as minimisation factors, most respondents felt that this was dependent on the size of the trial. However, suggested rules of thumb included that only known (evidence-based) prognostic variables should be used and more than 10 is generally seen to be too many, with more than five or six variables making it tricky to test the programming. Nine respondents would not set a limit on the number of factors, and 13 would set a limit of four variables or less.

Sixty-six per cent of those who had used minimisation adjusted for the minimisation factors using covariate adjustment as appropriate (40% always, 25% sometimes) through analysis of covariance (ANCOVA), multiple regression, logistic regression or Cox's regression. There was a suggestion to perform both unadjusted and unadjusted analyses.

The most common problem encountered when using minimisation was programming difficulty or errors, and a few respondents stressed the importance of finding appropriate purpose-written software. It was observed that overall treatment imbalance can result if stratifying by centre or clinician, and two respondents mentioned checking for balance.

### Use of other forms of constrained randomisation

Respondents were asked about their use of other (less common) randomisation methods, including those of Atkinson [11], Titterington [12], Signorini and colleagues [13] and Klotz [14]. Forty-nine (42%) respondents had heard of Atkinson’s method but only one person had ever used it, saying the method did not work and they would not use it again. Although 25 (21%) respondents had heard of Titterington’s method and 13 (11%) had heard of Klotz’s method, no-one had ever used them. Of the 22 (19%) people who had heard of Signorini’s method, four had used the method and it had worked but two would not use the method again because they perceived it to be not as good as minimisation and not as easy to use.

### Opinions on choosing a randomisation method

The most important factor when choosing a randomisation method was seen as the size of the trial. The general advice was to keep it simple and the most common problem was identification of prognostic factors and limiting their number. Other important factors included the practicality of the method, maintaining blindness, importance of prognostic factors, number of centres, size of trial, length of trial and reducing predictability.

Ninety-eight (83%) respondents replied to the open text question about giving general advice to someone choosing a randomisation scheme. The five most common themes are listed in Table 3.

**Table 3:** General advice when choosing a randomisation scheme

	**2003**	**2011**	**Total**
	(***n***= **103**)	(***n***= **15**)	(***n***= **118**)
Keep it simple	31 (30%)	4 (27%)	35 (30%)
Consider important prognostic factors only (keep to a minimum)	24 (23%)	7 (47%)	31 (26%)
Limit predictability	10 (10%)	2 (13%)	12 (10%)
Speak to an experienced statistician/expert	9 (9%)	0 (0%)	9 (8%)
Use minimisation if possible	8 (8%)	3 (20%)	11 (9%)

Data presented as *n* (%).

### Problems encountered when setting up randomisation schemes

Eighty-nine (75%) respondents replied to the open text question outlining the most common problems encountered when setting up randomisation schemes (Table 4).

**Table 4:** Problems encountered when setting up randomisation schemes

	**2003**	**2011**	**Total**
	(***n*** = **103**)	(***n*** = **15**)	(***n*** = **118**)
Identification of/limiting prognostic factors/strata	11 (11%)	4 (27%)	15 (13%)
No problems	10 (10%)	2 (13%)	12 (10%)
Maintaining blindness/allocation concealment	10 (10%)	0 (0%)	10 (8%)
Costs of/problems with commercial services/telephone randomisation	6 (6%)	0 (0%)	6 (5%)
Ignorance or human mistakes	5 (5%)	1 (7%)	6 (5%)
Testing and monitoring	5 (5%)	1 (7%)	6 (5%)
Problems with/unavailable computer programs	5 (5%)	0 (0%)	5 (4%)
Patients randomised out of order/misallocation	5 (5%)	0 (0%)	5 (4%)
Drug supplies/stock management	3 (3%)	1 (7%)	4 (3%)

Data presented as *n* (%).

### Web-based randomisation

In 2003, 80% of respondents had never used Web-based randomisation systems although they would possibly use them in the future. In 2011 only one respondent had never used them and 11 (73%) had used them several times. The biggest problems were thought to be lack of expertise and security. Other problems to be overcome include access to the Web, speed and reliability of access, lack of willingness in centres, telephone methods being preferred and trust in those performing the randomisation.

### Future directions

Most respondents thought that minimisation is likely to be the most popular randomisation method in the future, with the use of a random element to avoid the possibility of prediction of treatment allocation, followed by permuted blocks. However, this was not a consensus view as the stated views of experts within the survey population ranged from ‘do not be misled by the hype surrounding minimisation – it is most emphatically NOT the platinum standard’ to ‘I would favour minimisation because of its advantages not just for small trials but also for large trials in which analysis within subgroups would be based on better balance’.

## Discussion

### Simple randomisation

The results of this survey have shown that trialists are currently using simple randomisation less often than permuted blocks, stratification and minimisation. The size of trial considered too small for simple randomisation ranged from <50 to <1,000, although some trialists surveyed believed that simple randomisation was suitable for any size of trial.

Within the wider literature, there is evidence that simple randomisation is suitable for larger trials [3,15-17] but there is a high probability of possible imbalance between treatment groups in trials of up to 500. Simulations showed that imbalance can occur often in small trials with <100 participants (for example, >80% of occasions), but imbalance rarely occurs in trials with ≥1,000 participants [15]. Pocock and Simon also suggested that simple randomisation was only to be recommended for larger trials (say >200 participants), and cautioned that even in large trials problems may occur if one intends to analyse early results (say for data monitoring) [16].

### Stratification

Forty-three per cent of trialists wrote that the number of strata to be used should depend on the size of trial. There was no general consensus on the number of strata that were considered too many, with suggested values between 1 and 100.

Pocock and Simon stated that it is seldom advisable to stratify on more than three or four variables and the size of the trial is the most important factor in deciding how many stratification variables are feasible [16]. Peto and colleagues dismiss stratification as a complication rendered unnecessary by the development of methods of analysis that adjust for covariates [18].

Pocock and Simon used simulations to show that increasing imbalance between treatment groups can arise due to increasing number of strata with incomplete randomised blocks [16]. Signorini and colleagues asserted that using a randomised block design presented a possibility of the overall trial having large differences in number of patients in each treatment, even though each stratum has more or less equal numbers on each treatment [13]. This leads to a loss in efficiency in the analysis. The same problem can arise when employing a randomisation scheme using minimisation with stratification by centre or clinician, although International Conference on Harmonisation (ICH) guidelines still recommend this [19].

### Balancing prognostic factors at baseline

More than one-half of the trialists surveyed thought that groups should be balanced at baseline. The primary reasons given were that it helps face-validity and the analysis is simpler. The suggested limit on the number of prognostic factors ranged from 2 to 10, and some trialists would not set any limit.

There is controversy, especially among statisticians, as to whether prognostic factors should be used in assignment to treatment [20]. There are two main viewpoints: prognostic factors should be used to assure that patients assigned to the two arms show close balance in prognoses at baseline; or random assignment should be used without regard to prognostic factors, as fair comparison of treatment effect can be achieved through statistical adjustment of results.

Armitage and Gehan concluded that in a small to moderate-sized trial (≤100 patients) results might be invalid if prognostic factors are not used [21]. Comparisons between treatments should be made between groups that are comparable with respect to prognostic factors. Similarly, Rovers and colleagues suggested that investigators should always consider balanced allocation for a low number of patients [22]. They stated that, in a trial with 100 to 200 patients, substantial differences can occur in baseline characteristics if simple randomisation is used; if these differences can be measured, then they can be corrected to some degree in the analysis. If the number of patients decreases or the number of prognostic variables or categories increases, then the resulting imbalance could invalidate results.

The Committee for Proprietary Medicinal Products state that stratification for more than a few prognostic factors is not always possible [23]. They describe techniques of dynamic allocation such as minimisation that are often used to balance across several factors simultaneously as highly controversial even if deterministic schemes are avoided. They therefore strongly advise against the use of such methods. When deterministic schemes are used, the Committee for Proprietary Medicinal Products also suggest that the factors used in the allocation scheme should also be included as covariates in the analysis (although whether the analysis adequately reflects the randomisation scheme also remains controversial). They also state that if a multicentre trial is not stratified by centre then the reasons for not doing this should be explained and justified in the protocol.

### Predictability

Nineteen per cent of trialists who had used minimisation were very concerned that the method was largely deterministic and 57% were only mildly concerned. Twenty-eight per cent were not concerned at all in 2003, but in 2011 this number had dropped to zero. Among the trialists surveyed, strategies for reducing the predictability of next assignment included not declaring the factors in the protocol, adding in other factors to produce noise, not stratifying by centre and alternating between minimisation and simple randomisation. When using a random element, the level of randomness chosen ranged from 0.66 to 0.95.

When choosing a balanced allocation method the probability of performing the prescribed allocation should be large enough to control imbalances in strata but small enough to prevent selection bias through prediction of next treatment [24]. The use of a random element (probability of assignment *P*<1) is supported by ICH E9 [19], which also states that factors on which randomisation has been stratified should be accounted for later in the analysis. Gore recommends a value for probability of assignment of 0.75 [25]. In a multicentre trial, predictability is not a problem unless stratifying by centre as recommended by ICH E9 guidelines, when all centres are essentially independent or they are not aware of the bands used [20].

The Food and Drug Administration also consider minimisation not to be random and recommend the incorporation of a random element [26]. However, a minimisation procedure to allocate treatment that does not allow investigators to predict the next treatment allocation would yield a properly randomised trial [16].

Randomised block design can also be highly predictable [13] since each block must contain equal numbers of patients on each treatment. The block size should be hidden from investigators but can sometimes be deduced from previous assignments if the block size is fixed. Therefore it is better to use random block sizes.

### Analysis

In 2003 almost one-quarter of those using minimisation never adjusted for prognostic factors in the analysis, believing that if the minimisation has worked then there should be no need to do so. In 2011 this number had dropped to zero. Forty per cent indicated that they always adjusted for the minimisation factors in the analysis.

Taves maintains that clinical trials managed by minimisation should use ANCOVA for statistical comparisons [10]. Lachin and colleagues indicate for minimisation that the statistical analysis must incorporate adjustments for the covariates employed in the design in order to yield tests of proper size [17].

Forsythe and Stitt studied analysis of variance and ANCOVA in a simulation study of a small clinical trial and found some evidence that ANCOVA with minimisation was more powerful than ANCOVA with simple randomisation [27]. They showed that using minimisation without adjusting for covariates in the subsequent analysis distorted the significance level and the power of the test. However, they reported that other studies implied that the effects of stratifying by a covariate and then ignoring it in the analysis may not be severe. It may be that introducing randomness into a systematic design reduces such accidental bias, but further research is needed.

Vaughan Reed and Wickham discussed the validity of using an unadjusted analysis [28]. They maintained that the errors introduced by using an unadjusted analysis for a deterministic allocation method were likely to be less than the errors resulting from imbalance between treatment groups that might occur using simple randomisation. They maintained that serious imbalance between treatment groups undoubtedly causes problems with analysis and interpretation.

### Strengths and limitations of the study

#### Sampling frame

For the original sampling frame, the Directory of Academic Statisticians is not compulsory so the list cannot claim to be exhaustive. The declared areas of interest are also voluntary and therefore relevant statisticians may have been missed using this source. Some research centres have dedicated programmers who set up randomisation schemes, and these people would not be included in the register. Although the second round of the survey did target these people, the response rate was very low – so it is difficult to draw any definite conclusions from these responses alone. Some individuals may also possibly have responded at both time points because the first survey was anonymous. Views from the pharmaceutical industry are not represented here at all.

#### Question formats

A mix of open-text and multiple-choice questions were included in the survey. Every effort was made to formulate the questions so as to elicit unbiased responses. However, some respondents queried the wording of some of the questions, perhaps reflecting the heated debate that was ongoing amongst clinical trialists, academics and governing bodies at the time the survey was sent out.

## Conclusions

From this survey it was apparent that if the number of important prognostic factors and layers within these is sufficiently small, then the preferred method of randomisation (agreed by most trialists and the ICH) is permuted blocks of varying random length within strata. This method reduces the problem of predictability and at the same time balances across combinations of factors. If the number of prognostic factors is large, then minimisation can be used to provide treatment balance as well as balance over these factors. However, only those factors known to affect outcome should be considered.

Trialists believe that keeping the randomisation method as simple as possible will reduce time, cost and programming errors. More complex algorithms may increase costs and errors, prove difficult to understand and may not prove to be less predictable. Whatever method is chosen, consideration should be given to using simulations to test the method first to ensure that the algorithm is correct, achieves balance and is not predictable.

Survey respondents stated that the method of randomisation should depend on the context of the study, the objectives of the study and the resources available. One method may not be suitable for all trials.

Survey respondents predict that minimisation will probably become more widely used in the future along with increased use of Web-based and telephone-based systems. However, very few respondents considered that more complex methods of randomisation offer any advantages.

## Abbreviations

ANCOVA: Analysis of covariance; ICH: International Conference on Harmonisation.

## Competing interests

The authors declare that they have no competing interests.

## Authors’ contributions

GCM and MKC conceived the idea for the study. GCM carried out the survey and analysis and wrote the first draft of the paper. GCM, MKC and DRE were all involved in the development and refinement of subsequent drafts. GCM is the guarantor for the study. All authors read and approved the final manuscript.

## Supplementary Material

Additional file 1 **Current use of statistical randomisation methods in clinical trials.** Survey instrument which was sent out in 2003 and 2011. Click here for file
