# Supplementary material for: Use of randomisation in clinical trials: a survey of UK practice
Source: Trials. 2012 Oct 26;13:198. doi: 10.1186/1745-6215-13-198 (PMC3522058; doi:10.1186/1745-6215-13-198)
Supplement: Additional file 1 — Current use of statistical randomisation methods in clinical trials. Survey instrument which was sent out in 2003 and 2011. [file 1745-6215-13-198-S1.doc]

# Appendix 1

# CURRENT USE OF STATISTICAL RANDOMIZATION METHODS IN CLINICAL TRIALS

| **1. Have you had any experience of setting up the randomization process for a clinical trial (eg organising the random number sequence, block generation, use of minimization etc)?** | Yes |  |
| --- | --- | --- |
|  |
|  | No |  |

If you answered No to Question 1 then this questionnaire is not relevant to you.

Please return it in the prepaid envelope provided or pass it on to a programmer, statistician or other researcher who has had experience of setting up the randomization process for a clinical trial.

Thank you for your time.

If you answered Yes to Question 1 please turn to page 2.

OPINIONS AND USE OF DIFFERENT STATISTICAL RANDOMIZATION METHODS

| **2. Which method(s) have you used to generate the random sequence?** *(please tick all that apply)* |
| --- |

| Simple randomization |  | Permuted blocks |  |  |  |
| --- | --- | --- | --- | --- | --- |
| Stratification |  | Minimization |  |  |  |

| Some other method(s) | |  | → Please describe |  |
| --- | --- | --- | --- | --- |
|  |  | | | |

| **3. Do you think that the use of simple randomization is appropriate for any size of trial, however small?** | Yes |  |
| --- | --- | --- |
|  |
|  | No |  |

| **If no, a) What size of trial do you consider too small for simple randomization?** |
| --- |

| Less than 50 |  | Less than 100 |  | Less than 500 |  |
| --- | --- | --- | --- | --- | --- |

| Other |  | → Please specify |  |
| --- | --- | --- | --- |

| **b) Which randomization method(s) would you use for these small trials?** | | |
| --- | --- | --- |
|  |  |  |
|  |  |  |

| **4. Many authors have indicated that if using stratification, one should not use “too many” strata. How many strata do you consider to be “too many”?** | | |
| --- | --- | --- |
|  |  |  |
|  |  |  |

| **5. Please give your opinions about the following two statements.** |
| --- |

| **a. Assignment of patients to treatment groups should take account of prognostic factors to ensure that the different treatments show close balance at baseline.** |
| --- |

| Do not agree | | |  | Agree |  | Unsure |  |
| --- | --- | --- | --- | --- | --- | --- | --- |
| Please give reasons | |  | | | | | |
|  |  | | | | | | |
| **b. Random assignment should be used without regard to prognostic factors. Fair comparison of treatment effect can be achieved through statistical adjustment of results.** | | | | | | | |

| Do not agree | | |  | Agree |  | Unsure |  |
| --- | --- | --- | --- | --- | --- | --- | --- |
| Please give reasons | |  | | | | | |
|  |  | | | | | | |

##### OPINIONS AND USE OF MINIMIZATION

| 1. **Have you ever used the method of minimization?**   **If no, please go straight to Q15.** | | | Yes | | |  | | |  | |
| --- | --- | --- | --- | --- | --- | --- | --- | --- | --- | --- |
|  | | |  | |
|  | | | No | | |  | | |  | |
|  | | **If yes, were there any difficulties/problems encountered either in setting up the method or arising throughout the randomization process?** | | | | | | | |  |
|  | |  | | | | | | | |  |
|  | |  | | | | | | | |  |
|  | |  | | | | | | | |  |
|  | |  | | | | | | | |  |
|  | |  | | | | | | | |  |
|  | |  | | | | | | | |  |
|  | |  | | | | | | | | |
|  | | **Did the method work?** | | | Yes | | |  | | |
|  | |  | | | No | | |  | | |
|  | |  | | | | | | | | |
|  | **Would you use it again?** | | | Yes | | |  | | |  |
|  |  | | | No | | |  | | |  |

| **7. When using minimization do you use the simple method described by Taves where there is no random element except in the case of ties (i.e. the probability of assignment to treatment group with smallest number of patients = 1) (Taves DR. Minimization: A new method of assigning patients to treatment and control groups. Clin Pharmacol Ther 1974;15:443-53.)?** |
| --- |

| Always |  | Sometimes |  | Never |  |
| --- | --- | --- | --- | --- | --- |

| **8. When using minimization do you ever weight any of the variables?** |
| --- |

| Always |  | Sometimes |  | Never |  |
| --- | --- | --- | --- | --- | --- |

| **9. Have you ever used either the “range” or “variance” methods of Pocock & Simon? (Pocock SJ, Simon R. Sequential treatment assignment with balancing for prognostic factors in the controlled clinical trial. *Biometrics* 1975;31:103-15.)** |
| --- |

| Several times |  | Once or twice |  | Never |  |
| --- | --- | --- | --- | --- | --- |

| **10. Have you ever been concerned that minimization can be largely deterministic (e.g. nonrandom)?** |
| --- |

| Very concerned |  | Mildly concerned |  | Not concerned at all |  |
| --- | --- | --- | --- | --- | --- |
| **11. Do you use any methods which attempt to reduce predictability while maintaining a balance between treatment groups?** | | | | | |

| Always |  | Sometimes |  | Never |  |
| --- | --- | --- | --- | --- | --- |

| If always or sometimes, please describe. | |  |
| --- | --- | --- |
|  |  | |
|  |  | |

| **12. Do you use any random element when using minimization (where the probability of allocation to treatment with fewer number of patients is not equal to 1)?** |
| --- |

| Always |  | Sometimes |  | Never |  |
| --- | --- | --- | --- | --- | --- |

| If always or sometimes, what level of randomness is chosen? |  |
| --- | --- |

| **Does this value change in response to the current status of the trial or is it fixed?** | |
| --- | --- |
|  |  |

| **13. When using minimization, how many factors and/or levels do you consider to be too many?** | |
| --- | --- |
|  |  |
|  |  |
|  |  |
|  |  |

| **14. Do you adjust for the minimization factors in the statistical analysis?** |
| --- |

| Always |  | Sometimes |  | Never |  |
| --- | --- | --- | --- | --- | --- |

| If always or sometimes, what method do you use? | |  |
| --- | --- | --- |
|  |  | |
|  |  | |

##### OTHER FORMS OF CONSTRAINED RANDOMIZATION

| **15. Some authors consider Atkinson's method to be a better alternative to minimization. Have you heard of Atkinson's method (Atkinson AC. Optimum biased coin designs for sequential clinical trials with prognostic factors. Biometrics 1982;69:61-67.)?** | Yes |  |
| --- | --- | --- |
|  | No |  |
|  |  |  |

| **If yes, have you ever used this method?** | Yes |  |
| --- | --- | --- |
|  | No |  |

|  | **If yes, were there any difficulties/problems encountered either in setting up the method or arising throughout the randomization process?** | | |
| --- | --- | --- | --- |
|  |  | | |
|  |  | | |
|  |  | | |
|  |  | | |
|  |  | | |
|  |  | | |
|  | | | |
| **Did the method work?** | | Yes |  |
|  | | No |  |
|  | | | |
| **Would you use it again?** | | Yes |  |
|  | | No |  |
|  | | | |
| **What kind of analysis was used?** | | | |
|  | | | |
|  | | | |

| **16. Have you heard of the minimum quadratic distance constrained balance constrained method of allocation by Titterington (Titterington DM. On constrained balance randomization for clinical trials. Biometrics 1983;39:1083-86.)?** | Yes |  |
| --- | --- | --- |
|  | No |  |

| **If yes, have you ever used this method?** | Yes |  |
| --- | --- | --- |
|  | No |  |

|  | **If yes, were there any difficulties/problems encountered either in setting up the method or arising throughout the randomization process?** | | | | |  |
| --- | --- | --- | --- | --- | --- | --- |
|  |  | | | | |  |
|  |  | | | | |  |
|  |  | | | | |  |
|  |  | | | | |  |
|  |  | | | | |  |
|  |  | | | | |  |
|  |  | | | | | |
|  | **Did the method work?** | | Yes | |  | |
|  |  | | No | |  | |
|  |  | | | | | |
| **Would you use it again?** | | Yes | |  | |  |
|  | | No | |  | |  |
|  | | | | | |  |
| **What kind of analysis was used?** | | | | | |  |
|  | | | | | |  |
|  | | | | | |  |

| **17. Have you heard of the constrained method of allocation by Klotz (Klotz JH. Maximum entropy constrained balance randomization for clinical trials. Biometrics 1978;34:283-87.)?** | Yes |  |
| --- | --- | --- |
|  | No |  |

| **If yes, have you ever used this method?** | Yes |  |
| --- | --- | --- |
|  | No |  |

|  | **If yes, were there any difficulties/problems encountered either in setting up the method or arising throughout the randomization process?** | | |
| --- | --- | --- | --- |
|  |  | | |
|  |  | | |
|  |  | | |
|  |  | | |
|  |  | | |
|  |  | | |
|  | | | |
| **Did the method work?** | | Yes |  |
|  | | No |  |
|  | | | |
| **Would you use it again?** | | Yes |  |
|  | | No |  |
| **What kind of analysis was used?** | | | |
|  | | | |
|  | | | |

| **18. Have you heard of Signorini's dynamic balanced randomization which balances the number of treatment allocations both within strata and in the overall trial, while retaining an element of randomness (Signorini DF, Leung O, Simes RJ, Beller E, Gebski VJ. Dynamic balanced randomization for clinical trials. Stat Med 1993;12:2343-50.)?** | Yes |  |
| --- | --- | --- |
|  | No |  |
|  |  |  |

| **If yes, have you ever used this method?** | Yes |  |
| --- | --- | --- |
|  | No |  |

|  | **If yes, were there any difficulties/problems encountered either in setting up the method or arising throughout the randomization process?** | | |
| --- | --- | --- | --- |
|  |  | | |
|  |  | | |
|  |  | | |
|  |  | | |
|  |  | | |
|  |  | | |
|  | | | |
| **Did the method work?** | | Yes |  |
|  | | No |  |
|  | | | |
| **Would you use it again?** | | Yes |  |
|  | | No |  |
|  | | | |
| **What kind of analysis was used?** | | | |
|  | | | |
|  | | | |

| **19. Have you used any other constrained randomization methods that are not mentioned here?** | | | Yes |  |
| --- | --- | --- | --- | --- |
|  |  |  | No |  |
|  |  |  |  |  |
| If yes, what are these? | |  | | |
|  |  | | | |
|  |  | | | |

|  | **What do you consider are the advantages/disadvantages of these methods? Please specify any problems encountered with their use.** | | |
| --- | --- | --- | --- |
|  |  | | |
|  |  | | |
|  |  | | |
|  |  | | |
|  | | | |
| **Did the method work?** | | Yes |  |
|  | | No |  |
|  | | | |
| **Would you use it again?** | | Yes |  |
|  | | No |  |
|  | | | |
| **What kind of analysis was used?** | | | |
|  | | | |
|  | | | |

##### OPINIONS ON CHOOSING A RANDOMIZATION METHOD

| **20. What do you consider to be important factors when choosing a randomization method?**  *(please indicate all that you consider to be important)* |
| --- |

| Size of trial |  | Number of prognostic factors |  | Number of strata |  |
| --- | --- | --- | --- | --- | --- |

| Cost |  | Complexity of method |  |
| --- | --- | --- | --- |

| Other | |  | → Please specify |  |
| --- | --- | --- | --- | --- |
|  |  | | | |

| **21. What general advice would you give to someone choosing a randomization scheme for a trial?** | |
| --- | --- |
|  |  |
|  |  |
|  |  |
|  |  |
|  |  |
|  |  |

| **22. What are the biggest or most common problems you have encountered when setting up randomization schemes?** | |
| --- | --- |
|  |  |
|  |  |
|  |  |
|  |  |
|  |  |
|  |  |
|  |  |

##### WEB-BASED RANDOMIZATION

| **23.** **Web based randomization is becoming increasingly popular. Have you ever used the Web for randomization?** |
| --- |

| Several times |  | Once or twice |  | Never |  |
| --- | --- | --- | --- | --- | --- |

| **If never, do you think that you will use it in the future?** |
| --- |

| Probably |  | Possibly |  | Definitely not |  |
| --- | --- | --- | --- | --- | --- |

| **24. What do you think are the biggest problems to be overcome in using the Web for randomization?** *(please indicate all that you consider to be important)* |
| --- |

| Cost |  | Security |  |
| --- | --- | --- | --- |

| Lack of technology |  | Lack of expertise |  |
| --- | --- | --- | --- |

| Other | |  | →Please specify |  |
| --- | --- | --- | --- | --- |
|  |  | | | |

| **25. What do you think will be the most popular randomization methods in the future?** |
| --- |
|  |
|  |
|  |
|  |
|  |
|  |

| **26. Any other comments.** |
| --- |
|  |
|  |
|  |
|  |
|  |
|  |
|  |
|  |
|  |
|  |
|  |
|  |
|  |
|  |
|  |
|  |

***Many thanks for the time you have spent completing this questionnaire. Please return it in the reply-paid envelope enclosed.***

***Your feedback is very important to us, and we appreciate your response.***

Mrs Gladys McPherson, Senior Trials Programmer

Health Services Research Unit, University of Aberdeen

Polwarth Building, Foresterhill

Aberdeen, AB25 2ZD

Telephone: 01224 554339

Email: [g.mcpherson@abdn.ac.uk](mailto:g.mcpherson@abdn.ac.uk)
